# Association between Hypertension and the Prevalence of Low Back Pain and Osteoarthritis in Koreans: A Cross-Sectional Study

**DOI:** 10.1371/journal.pone.0138790

**Published:** 2015-09-22

**Authors:** Young-Hyeon Bae, Joon-Shik Shin, Jinho Lee, Me-riong Kim, Ki Byung Park, Jae-Heung Cho, In-Hyuk Ha

**Affiliations:** 1 Jaseng Spine and Joint Research Institute, Jaseng Medical Foundation, Seoul, Republic of Korea; 2 Department of Korean Rehabilitation Medicine, Kyung Hee University, Seoul, Republic of Korea; Hospital de Clínicas de Porto Alegre, BRAZIL

## Abstract

**Background:**

Hypertension and musculoskeletal disorders are highly prevalent in adult populations. The objective of this study was to investigate the association between hypertension and prevalence of low back pain (LBP) and osteoarthritis in Koreans.

**Methods:**

A total 17,128 participants (age ≥20 years) who answered low back pain and osteoarthritis items in the 4th Korean National Health and Nutrition Examination Survey (2007–2009) were analyzed. Odds ratios were calculated using logistic regression and were adjusted for age, sex, income level, education, occupation, BMI, smoking status, alcohol consumption, and physical activity.

**Results:**

Lifetime prevalence of LBP in hypertensive subjects was 34.4%, and that of osteoarthritis 26.2%. LBP prevalence was significantly lower in hypertensives (fully adjusted OR 0.79; 95% CI 0.70–0.90), and both LBP and osteoarthritis prevalence was significantly lower in participants with systolic blood pressure ≥140mmHg than those with <120mmHg (fully adjusted OR 0.81; 95% CI 0.70–0.94, and 0.81; 95% CI 0.68–0.96, respectively). Prevalence of LBP in subjects with diastolic blood pressure ≥90mmHg was also significantly lower than those with <80mmHg (fully adjusted OR 0.73; 95% CI 0.63–0.85). LBP and osteoarthritis prevalence did not differ by systolic or diastolic blood pressure interval in respondents taking antihypertensive medication. LBP and osteoarthritis prevalence increased with longer hypertension duration (fully adjusted p for trend 0.028, and 0.0008, respectively).

**Conclusions:**

Hypertension showed an inverse relationship with LBP and osteoarthritis prevalence, which may be ascribed to hypertension-associated hypalgesia, and antihypertensive medication intake and longer hypertension duration attenuated this association.

## Introduction

Low back pain (LBP) continues to be one of the most common musculoskeletal disorders causing disability, severe pain, and extended sick leave at substantial personal and social expense [1], and an estimated 70–80% of adults suffer from LBP at some point of their lives [2]. Osteoarthritis (OA) is the most common form of arthritis in western countries with significant impact on morbidity, quality of life and healthcare costs. Osteoarthritis is a common end stage phenotype of multifactorial etiology involving joint tissue as opposed to a single disease entity [3]. Some factors contributing to the increase of osteoarthritis are aging populations, and insufficient treatment to cope with or alter the course of this health condition [4].

Cardiovascular disease risk factors such as obesity, smoking, and serum lipid levels have been implicated with LBP and osteoarthritis through the atherosclerosis hypothesis. Low-grade systemic inflammation is present in obesity, and most obese people display elevated inflammatory markers including C-reactive protein [5–7], while smoking decreases bone mineral content, leading to osteoporosis and higher susceptibility to microfractures in the trabecular bone of the vertebral bodies [8], possibly eliciting pain. There are also reports that high serum cholesterol and triglyceride levels result in higher LBP occurrence [9].

Contrary to other major cardiovascular disease risk factors, hypertension has been shown to be inversely associated with LBP and osteoarthritis in several studies. This seems to denote hypalgesic mechanism involvement, with a growing body of evidence supporting the hypertension-related hypalgesia theory associating elevated blood pressure with higher pain thresholds: Increase in blood pressure levels was reported to be associated with hypalgesic mechanism in the relationship between pain perception and blood pressure [10], and difference in plasma beta endorphin levels in hypertensives and normotensives points to a relationship with endogenous opioids. Animal studies in cats and rats have shown that pain thresholds increase in hypertensive groups compared to normotensives, and production of antinociceptions also supports hypertension-related hypalgesia [11]. However, definite conclusions have not been produced [12–14], and owing to conflicting results in a paucity of definitive studies on the relationship between hypertension, LBP and osteoarthritis, it is still unclear which factors are related with hypertension-associated hypalgesia [15–19].

The objective of this epidemiological research was to conduct a large-scale cross-sectional study at the population level using the Korean National Health and Nutrition Examination Survey (KNHANES) data, adjusting for relevant covariates in assessing the association of hypertension with pain sensitivity in LBP and osteoarthritis. We further investigated the association of antihypertensive drugs and hypertension duration period with sensitivity to pain in this sample.

## Materials and Methods

### Study population and sampling

This study analyzed data from the KNHANES IV (2007–2009) which used a stratified, multistage, probability-cluster sampling method in a rolling sampling survey of South Korean citizens. The survey was conducted by the Korean Ministry of Health and Welfare and had 3 sections—a health survey, health consultation, and nutrition survey. Additional information is available on the KNHANES website in “The 4^th^ KNHANES Sample Design” and 1^st^-3^rd^ Sample Design reports [20]. The KNHANES data is available through email request. A total 23,632 subjects completed the KNHANES IV (2007–2009) health examination and questionnaire (74.5% of target population; target population n = 31,705). Analysis was limited to results of the 17,128 adults aged ≥20 years who answered the LBP and osteoarthritis survey items.

### Low back pain

Lifetime LBP was defined as any previous experience of LBP reported in the health questionnaire (“Have you had any previous experience of LBP?”), and chronic LBP as pain episodes persisting ≥3 months during the previous year (“Have you experienced LBP for 3 months or longer during the previous year?”).

### Osteoarthritis

The definition of lifetime osteoarthritis was any previous experience of osteoarthritis reported in the health survey (“Have you had any previous experience of osteoarthritis?”), and that of chronic osteoarthritis, self-reported osteoarthritis accompanying pain symptoms lasting ≥3 months during the previous year (“Have you experienced osteoarthritis for 3 months or longer during the previous year?”).

### Hypertension

Blood pressure was measured 3 times by trained nurses using a mercury sphygmomanometer with 25–35 cm cuff (Baumanometer, WA Baum Co., NY, USA) in a seated position with arm supported at heart level after a 5 minute rest. Measurements were recorded to the closest 2 mmHg on the manometer, and analyzed using the average of the 2^nd^ and 3^rd^ measurements. The nurses and examiners were trained 2–4 weeks prior to task assignment, and received regular refresher training (7 sessions per year) during operation. hypertension was classified according to the KNHANES criteria, which defines hypertension as a systolic blood pressure (SBP) of ≥140 mmHg or diastolic blood pressure (DBP) of ≥90 mmHg or regular use of antihypertensive medication, prehypertension as a SBP of ≥120 mmHg and <139 mmHg or DBP of ≥80 and <89, and normal blood pressure as a SBP of <120 mmHg and DBP of <80 mmHg.

### Covariates

Age, sex, income level, education level, occupation, body mass index (BMI), smoking status, alcohol consumption, and moderately exerting physical activity were included as general, socioeconomic, and lifestyle-related characteristics of the participants. Income level was classified into quartile ranges by monthly average household income adjusted with equalization (monthly household income divided by total number of household members). Education level was grouped as elementary school graduation or lower, middle school graduation, high school graduation, and college graduation or higher. Occupation was regrouped from the 6th revision of the Korean Standard Classification of Occupation into 7 groups: (a) manager, administrator, or professional worker, (b) office worker, (c) retail or service industry worker, (d) agriculture or fisheries worker, (e) machine manufacturer or operator, (f) manual worker, and (g) unemployed. Information on smoking habits, alcohol consumption, and physical activity were also collected. BMI (kg/m^2^) was classified into 3 categories (<18.5, <25, and ≥25) by physical measurements. Smoking had 3 categories: (a) having smoked a total ≥5 packs of cigarettes and current smoking was defined as ‘current smokers’; (b) having smoked a total ≥5 packs of cigarettes and current nonsmoking was defined as ‘exsmokers’; and (c) those with <5 packs of cigarette smoking as ‘never smokers’. Alcohol consumption was divided into 2 groups: (a) ‘non-alcohol consumers’ with no alcohol consumption for the past year or consumption <once a month; and (b) ‘alcohol consumers’ with alcohol consumption ≥once a month. Level of physical activity in leisure time was classified into 2 categories with (a) ‘regular physical exercise’ comprising of ≥one 30 minute session of moderately strenuous physical exercise or with slightly labored breathing (low intensity swimming, doubles tennis, volleyball, etc) over the past week, and (b) ‘non-regular physical exercise’ as beneath this level.

### Statistical analysis

The KNHANES applies stratified cluster sampling and weighted values to a nationally representative sample. We also conducted data analysis using complex sampling design considering such factors as stratified variables, cluster variables, and weighted variables, and all data analysis was performed with the statistical software package SAS version 9.3 (SAS Institute Inc, Cary, NC, USA), and *P* < 0.05 was considered to be statistically significant. Continuous variables were calculated as mean and standard deviation, and categorical variables evaluated as frequency and percentage (%). Difference in characteristics by LBP and osteoarthritis status was assessed with the Rao-Scott Chi-Square test or t-test as appropriate. hypertension-related factors were analyzed for association with LBP and osteoarthritis using logistic regression analysis for complex sampling adjusted for select variables, and are presented as odds ratios (OR) with 95% confidence intervals (CI). We also examined linear associations by duration period in each model (p for trend) in assessment of hypertension duration, and compared fully adjusted and selectively adjusted estimates for sensitivity analysis using backward elimination in stepwise regression.

### Ethics statement

The survey interviewer was not given prior information about a participant before performing the interview, and all respondents provided written informed consent to participate. The study was approved by the Institutional Review Board of Jaseng Hospital of Korean Medicine in Seoul, Korea in accordance with the Declaration of Helsinki.

## Results

Of 31,705 subjects invited to participate in KNHANES IV, 17,128 subjects aged 20 years or older who completed the LBP and osteoarthritis sections were available for analysis out of 23,632 participants (participation rate 74.5% of target population) who responded to the health-related questionnaire and examination.

The lifetime prevalence of LBP in Koreans aged ≥20 years was 28.8% (males 9.0%; females 19.8%), and that of osteoarthritis 16.3% (males 3.8%, females 12.5%). The LBP and osteoarthritis prevalence in hypertensive populations was 34.4% and 26.2%, respectively.

We included age, sex, socioeconomic variables (household income, education, occupation), and lifestyle risk factors (BMI, smoking status, alcohol consumption, regular moderate-intensity exercise) as confounding variables in assessing the difference in LBP and osteoarthritis prevalence within hypertensive populations. Prevalence of LBP and osteoarthritis all showed statistically significant differences (Table 1).

**Table 1:** Characteristics of Korean Surveyees of KNHANES IV aged ≥20 years (N = 17,128).

	LBP			OA		
	No (n = 12,207)	Yes (n = 4,921)	*P* ^b^	No (n = 14,335)	Yes (n = 2,793)	*P* ^b^
Age (mean (SD))^a^	43.2 (15.0)	50.6 (16.7)	<0.0001	42.8 (14.8)	59.9 (13.6)	<0.0001
Sex^a^						
Male	5,715 (78.8)	1,540 (21.2)	<0.0001	6,601 (91.0)	654 (9.0)	<0.0001
Female	6,492 (65.8)	3,381 (34.2)		7,734 (78.3)	2,139 (21.7)	
Household income^a^						
Low	2,934 (70.3)	1,239 (29.7)	0.1014	3,415 (81.8)	758 (18.2)	0.0469
Lower middle	2,870 (69.1)	1,283 (30.9)		3,478 (83.8)	675 (16.3)	
Higher middle	3,022 (72.3)	1,160 (27.7)		3,544 (84.7)	638 (15.3)	
High	3,039 (72.6)	1,148 (27.4)		3,558 (85.0)	629 (15.0)	
Education^a^						
≤Elementary school	2,824 (56.1)	2,210 (43.9)	<0.0001	3,215 (63.9)	1,819 (36.1)	<0.0001
Middle school	1,355 (70.5)	568 (29.5)		1,550 (80.6)	373 (19.4)	
High school	4,438 (77.4)	1,297 (22.6)		5,335 (93.0)	400 (7.0)	
≥College	3,528 (80.7)	844 (19.3)		4,178 (95.6)	194 (4.4)	
Occupation^a^						
Manager, administrator, or professional worker	1,540 (82.4)	330 (17.7)	<0.0001	1,787 (95.6)	83 (4.4)	<0.0001
Office worker	1,011 (83.6)	199 (16.5)		1,158 (95.7)	52 (4.3)	
Retail or service industry worker	1,579 (76.0)	499 (24.0)		1,856 (89.3)	222 (10.7)	
Agriculture or fisheries worker	856 (52.5)	775 (47.5)		1,186 (72.7)	445 (27.3)	
Machine manufacturer or operator	1,227 (77.8)	350 (22.2)		1,438 (91.2)	139 (8.8)	
Manual worker	1,008 (69.0)	453 (31.0)		1,169 (80.0)	292 (20.0)	
Unemployed	3,712 (64.6)	2,031 (35.4)		4,495 (78.3)	1,248 (21.7)	
Smoking status^a^						
Never smoker	7,035 (67.8)	3,346 (32.2)	<0.0001	8,315 (80.1)	2,066 (19.9)	<0.0001
Exsmoker	2,178 (73.6)	782 (26.4)		2,573 (86.9)	387 (13.1)	
Current smoker	2,946 (79.1)	779 (20.9)		3,392 (91.1)	333 (8.9)	
Body mass index^a^						
<18.5	593 (76.1)	186 (23.9)	0.0044	725 (93.1)	54 (6.9)	<0.0001
<25	7,773 (71.8)	3,048 (28.2)		9,329 (86.2)	1,492 (13.8)	
≥25	3,765 (69.4)	1,663 (30.6)		4,204 (77.5)	1,224 (22.6)	
Alcohol consumption^a^						
No	6,129 (65.0)	3,294 (35.0)	<0.0001	7,308 (77.6)	2,115 (22.5)	<0.0001
Yes	6,032 (78.9)	1,617 (21.1)		6,978 (91.2)	671 (8.8)	
Exercise^a^						
No	10,524 (72.0)	4,094 (28.0)	0.0008	12,288 (84.1)	2,330 (15.9)	0.0347
Yes	1,596 (66.3)	810 (33.7)		1,956 (81.3)	450 (18.7)	
Regular use of Antihypertensive medication						
No	10,317 (73.5)	3,718 (26.5)	<0.0001	12,254 (87.3)	1,781 (12.7)	<0.0001
Yes	1,834 (60.8)	1,183 (39.2)		2,027 (67.2)	990 (32.8)	
Intake of antihypertensive medication on day of survey						
No	12,038 (71.3)	4,850 (28.7)	0.3199	14,177 (83.9)	2,711 (16.1)	<0.0001
Yes	168 (70.3)	71 (29.7)		157 (65.7)	82 (34.3)	
SBP (mean (SD))	116.1 (16.5)	118.2 (17.8)	<0.0001	115.6 (16.4)	123.8 (18.3)	<0.0001
DBP (mean (SD))	76.9 (11.0)	75.6 (10.7)	<0.0001	76.5 (11.0)	77.5 (10.4)	0.0001

DBP, diastolic blood pressure; KNHANES, Korean National Health and Nutrition Examination Survey; SBP, systolic diastolic blood pressure; SD, standard deviation.

^a^ Covariates included for adjustment.

^b^ *P*-value of t-test or Rao-scott chi-square test for continuous and categorical variables.

The adjusted OR of LBP prevalence was significantly lower than normotensive subjects in hypertensives (fully adjusted OR 0.79; selectively adjusted OR 0.79). However, the adjusted OR of osteoarthritis occurrence was not significant for either fully adjusted or selectively adjusted estimates (Table 2).

**Table 2:** Associations Between Blood Pressure Levels and Low Back Pain or Osteoarthritis in Korean Surveyees of KNHANES IV aged ≥20 years^a^.

		Crude			Adjusted for age and sex			Fully adjusted^b^			Selectively adjusted^c^		
	N (case)	OR	95% CI	*P*	OR	95% CI	*P*	OR	95% CI	*P*	OR	95% CI	*P*
Low back pain^d^													
Normal	7,804 (2,035)	1.00			1.00			1.00			1.00		
Prehypertension	4,165 (1,124)	1.00	0.90, 1.12	0.9821	0.93	0.82, 1.04	0.1969	0.89	0.78, 1.01	0.0598	0.88	0.78, 1.00	0.0471
Hypertension	5,060 (1,740)	1.36	1.23, 1.50	<0.0001	0.85	0.76, 0.95	0.0045	0.79	0.70, 0.90	0.0002	0.79	0.69, 0.89	0.0001
Osteoarthritis^e^													
Normal	7,804 (780)	1.00			1.00			1.00			1.00		
Prehypertension	4,165 (663)	1.58	1.39, 1.79	<0.0001	1.18	1.02, 1.36	0.0219	0.97	0.83, 1.13	0.6868	1.00	0.87, 1.16	0.9736
Hypertension	5,060 (1,325)	3.33	2.96, 3.73	<0.0001	1.24	1.09, 1.42	0.0013	0.96	0.83, 1.11	0.5400	0.97	0.85, 1.12	0.7064

CI, confidence interval; KNHANES, Korean National Health and Nutrition Examination Survey; OR, odds ratio.

^a^ Hypertension diagnosis was made when patients met international standards (SBP≥140 mmHg or DBP≥90 mmHg) or were already on medication.

^b^ Adjusted for age, sex, household income, education, occupation, BMI, smoking, alcohol consumption and exercise patterns.

^c^ Adjusted for age, sex, education, occupation, BMI, alcohol consumption and exercise patterns in low back pain, and age, sex, education, BMI and exercise patterns in osteoarthritis. Backward elimination method was used with *P*<0.05 regarded to be significant.

^d^ Lifetime low back pain: any previous experience of low back pain.

^e^ Lifetime osteoarthritis: any previous experience of osteoarthritis.

LBP prevalence was significantly lower than subjects with a SBP of <120 mmHg in those with ≥140 mmHg (fully adjusted OR 0.81; selectively adjusted OR 0.84), and in those with a DBP of ≥90 mmHg compared to those with <80 mmHg (fully adjusted OR 0.73; selectively adjusted OR 0.73). While osteoarthritis prevalence displayed similar results with lower occurrence in subjects with a SBP of ≥140 mmHg than those with <120 mmHg (fully adjusted OR 0.81; selectively adjusted OR 0.79), no significant difference was found in subjects with a DBP of ≥90 mmHg (Table 3).

**Table 3:** Associations Between Systolic and Diastolic Blood Pressure and Low Back Pain or Osteoarthritis in Korean Surveyees of KNHANES IV aged ≥20 years^a^.

			Crude			Adjusted for age and sex			Fully adjusted^b^			Selectively adjusted^c^		
		N (case)	OR	95% CI	*P*	OR	95% CI	*P*	OR	95% CI	*P*	OR	95% CI	*P*
Low back pain^d^														
SBP	<120	9,876 (2,574)	1.00			1			1			1		
	<140	5,118 (1,572)	1.19	1.08, 1.30	0.0003	0.94	0.85, 1.04	0.2449	0.9	0.80, 1.00	0.0571	0.92	0.82, 1.02	0.1077
	≥140	2,110 (773)	1.54	1.35, 1.77	<0.0001	0.89	0.77, 1.02	0.0881	0.81	0.70, 0.94	0.0051	0.84	0.73, 0.97	0.0189
DBP	<80	10,536 (3,101)	1.00			1			1.00			1		
	<90	4,464 (1,277)	0.92	0.83, 1.02	0.1094	0.9	0.81, 1.00	0.0465	0.86	0.77, 0.97	0.0105	0.86	0.76, 0.96	0.0082
	≥90	2,104 (541)	0.81	0.71, 0.93	0.0025	0.79	0.69, 0.91	0.0011	0.73	0.63, 0.85	<0.0001	0.73	0.63, 0.85	<0.0001
Osteoarthritis^e^														
SBP	<120	9,876 (1,137)	1.00			1			1			1		
	<140	5,118 (1,083)	2.01	1.81, 2.24	<0.0001	1.1	0.97, 1.25	0.1233	0.91	0.79, 1.04	0.1701	0.94	0.83, 1.06	0.2979
	≥140	2,110 (569)	3.11	2.71, 3.57	<0.0001	0.93	0.79, 1.09	0.3787	0.81	0.68, 0.96	0.0178	0.79	0.67, 0.93	0.0056
DBP	<80	10,536 (1,624)	1			1			1.00			1		
	<90	4,464 (822)	1.24	1.11, 1.39	0.0001	1.14	1.01, 1.28	0.0352	0.97	0.84, 1.11	0.6071	0.98	0.87, 1.11	0.7682
	≥90	2,104 (343)	1.14	0.97, 1.33	0.1073	1.08	0.91, 1.28	0.3968	0.85	0.71, 1.01	0.0628	0.88	0.74, 1.05	0.1629

CI, confidence interval; DBP, diastolic blood pressure; KNHANES, Korean National Health and Nutrition Examination Survey; OR, odds ratio; SBP, systolic diastolic blood pressure.

^a^ Individuals with SBP and DBP measurements

^b^ Adjusted for age, sex, household income, education, occupation, BMI, smoking, alcohol consumption and exercise patterns.

^c^ Adjusted for age, sex, education, occupation, alcohol consumption and exercise patterns in association between SBP and low back pain, and age, sex, education, occupation, BMI, alcohol consumption and exercise patterns in that between DBP and low back pain. Adjusted for age, sex, education, BMI and exercise patterns in associations between SBP and DBP with osteoarthritis. Backward elimination method was used with *P*<0.05 regarded to be significant.

^d^ Lifetime low back pain: any previous experience of low back pain.

^e^ Lifetime osteoarthritis: any previous experience of osteoarthritis

A total 3,014 participants who took antihypertensive medication ≥15 doses per month or had taken antihypertensive medication on the day of the survey did not differ in LBP or osteoarthritis prevalence by level of SBP or DBP (Table 4).

**Table 4:** Associations Between Systolic and Diastolic Blood Pressure and Low Back Pain or Osteoarthritis in Korean Surveyees of KNHANES IV with Hypertensive Drug Use aged ≥20 years^a^.

			Crude			Adjusted for age and sex			Fully adjusted^b^			Selectively adjusted^c^		
		N (case)	OR	95% CI	*P*	OR	95% CI	*P*	OR	95% CI	*P*	OR	95% CI	*P*
Low back pain^d^														
SBP	<120	673 (257)	1.00			1.00			1.00			1		
	<140	1,438 (539)	0.95	0.76, 1.19	0.6666	0.91	0.72, 1.15	0.4154	0.9	0.69, 1.17	0.44	0.91	0.70, 1.19	0.4996
	≥140	903 (383)	1.22	0.93, 1.58	0.1487	1.05	0.79, 1.39	0.752	0.93	0.69, 1.26	0.6556	0.98	0.73, 1.31	0.8753
DBP	<80	1,376 (583)	1.00			1.00			1.00			1		
	<90	1,075 (403)	0.73	0.60, 0.90	0.0027	0.98	0.79, 1.22	0.8828	0.89	0.71, 1.12	0.3316	0.93	0.74, 1.17	0.5292
	≥90	563 (383)	0.66	0.51, 0.85	0.0016	1.07	0.82, 1.40	0.6327	0.96	0.72, 1.29	0.804	1.01	0.75, 1.35	0.9615
Osteoarthritis^e^														
SBP	<120	673 (232)	1.00			1.00			1.00			1		
	<140	1,438 (446)	0.84	0.67, 1.04	0.1086	0.78	0.61, 0.98	0.036	0.73	0.56, 0.96	0.0257	0.73	0.57, 0.93	0.0102
	≥140	903 (306)	0.95	0.75, 1.21	0.6792	0.78	0.61, 1.00	0.0532	0.77	0.58, 1.02	0.0693	0.73	0.56, 0.96	0.0237
DBP	<80	1,376 (497)	1.00			1.00			1.00			1		
	<90	1,075 (345)	0.76	0.62, 0.93	0.0077	1.06	0.85, 1.32	0.6301	0.95	0.74, 1.22	0.6697	1	0.80, 1.26	0.9972
	≥90	563 (142)	0.55	0.43, 0.70	<0.0001	0.9	0.69, 1.18	0.4301	0.78	0.58, 1.05	0.0951	0.82	0.62, 1.08	0.1566

CI, confidence interval; DBP, diastolic blood pressure; KNHANES, Korean National Health and Nutrition Examination Survey; OR, odds ratio; SBP, systolic diastolic blood pressure.

^a^ Individuals with hypertensive medicine use of ≥15 doses per month or hypertensive medicine use on day of survey (N = 3014).

^b^ Adjusted for age, sex, household income, education, occupation, BMI, smoking, alcohol consumption and exercise patterns.

^c^ Adjusted for age, sex, education, occupation and BMI in low back pain, and age, sex, education and BMI in osteoarthritis. Backward elimination method was used with *P*<0.05 regarded to be significant.

^d^ Lifetime low back pain: any previous experience of low back pain.

^e^ Lifetime osteoarthritis: any previous experience of osteoarthritis.

LBP prevalence by hypertension duration period could be inferred in 3,167 respondents who gave information on time of hypertension diagnosis (age at time of hypertension diagnosis subtracted from age at time of survey) (Table 5). LBP prevalence increased in subjects with a longer duration period of hypertension (fully adjusted p for trend 0.028; selectively adjusted p for trend 0.0383), and that of osteoarthritis also (fully adjusted p for trend 0.0232; selectively adjusted p for trend 0.0008). Hypertension patients who had been diagnosed >10 years earlier showed statistically higher LBP prevalence than patients diagnosed ≤3 years previous (fully adjusted OR 1.42; selectively adjusted OR 1.39), and osteoarthritis prevalence also (fully adjusted OR 1.42; selectively adjusted OR 1.54).

**Table 5:** Associations Between Hypertension Duration Period and Low Back Pain or Osteoarthritis in Korean Surveyees of KNHANES IV aged ≥20 years^a^.

		Crude			Adjusted for age and sex			Fully adjusted^b^			Selectively adjusted^c^		
	N (case)	OR	95% CI	*P*	OR	95% CI	*P*	OR	95% CI	*P*	OR	95% CI	*P*
Low back pain^d^													
HTN duration period^e^												
≤3 years	1,156 (397)	1.00			1.00			1.00			1		
≤5 years	547 (212)	1.34	1.05, 1.72	0.0202	1.22	0.94, 1.58	0.1427	1.18	0.88, 1.57	0.2781	1.19	0.90, 1.59	0.2266
≤10 years	765 (296)	1.31	1.04, 1.64	0.0205	1.17	0.92, 1.48	0.2053	1.16	0.88, 1.52	0.2894	1.14	0.88, 1.48	0.3272
>10 years	699 (312)	1.78	1.39, 2.28	<0.0001	1.25	0.96, 1.63	0.104	1.42	1.05, 1.91	0.0211	1.39	1.04, 1.86	0.0286
*P* for trend				<0.0001			0.0972			0.028			0.0383
Osteoarthritis^f^													
HTN duration period^e^													
≤3 years	1,156 (318)	1.00			1.00			1.00			1		
≤5 years	547 (156)	1.07	0.81, 1.42	0.6289	0.93	0.69, 1.27	0.6686	0.89	0.65, 1.23	0.4753	0.92	0.68, 1.26	0.6184
≤10 years	765 (237)	1.43	1.11, 1.84	0.0051	1.29	0.97, 1.70	0.0795	1.09	0.79, 1.50	0.5854	1.24	0.93, 1.65	0.1519
>10 years	699 (292)	2.3	1.85, 2.86	<0.0001	1.61	1.27, 2.04	<0.0001	1.42	1.08, 1.86	0.0125	1.54	1.20, 1.97	0.0007
*P* for trend				<0.0001			<0.0001			0.0232			0.0008

CI, confidence interval; KNHANES, Korean National Health and Nutrition Examination Survey; OR, odds ratio.

^a^ Individuals with data for time of HTN diagnosis (N = 3167)

^b^ Adjusted for age, sex, household income, education, occupation, BMI, smoking, alcohol consumption and exercise patterns.

^c^ Adjusted for age, sex, education, occupation and BMI in low back pain, and age, sex, education and BMI in osteoarthritis. Backward elimination method was used with *P*<0.05 regarded to be significant.

^d^ Lifetime low back pain: any previous experience of low back pain.

^e^ Duration period was classified as quartiles.

^f^ Lifetime osteoarthritis: any previous experience of osteoarthritis.

To provide some referential statistics, the prevalence of chronic LBP is 15.9% (males 4.5%, females 11.4%), and that of chronic osteoarthritis 12.6% (males 2.6%, females 10.0%) in Koreans aged ≥20 years. The difference in chronic LBP and osteoarthritis prevalence by blood pressure was comparable to that of lifetime LBP and osteoarthritis, with hypertension or higher levels of SBP and DBP showing an inverse association with chronic LBP and osteoarthritis prevalence and antihypertensive medication attenuating this relationship. Meanwhile, hypertension duration period had low correlations with chronic LBP and chronic osteoarthritis prevalence (see S1 File, which presents associations between blood pressure and chronic LBP and chronic osteoarthritis prevalence).

## Discussion

This study demonstrated an inverse relationship between high blood pressure and LBP and osteoarthritis prevalence. Antihypertensive medication intake attenuated this relationship through pain sensitivity modulation, and lower pain sensitivity was found in shorter duration of hypertension, i.e. more acute onset of hypertension. The study sample was representative of Korean men and women aged 20 and older. These results are consistent with previous studies accounting the inverse relationship between hypertension and chronic musculoskeletal complaints such as chronic LBP, which also reported that increased blood pressure was associated with diminished pain sensitivity in chronic musculoskeletal disorders [21,22].

There have been various studies investigating the association between hypertension and pain conditions. An occupational cohort based in the U.K. found no evidence of associations between hypertension and LBP [23]. Also, a cross-sectional study assessing musculoskeletal complaints in 1,858 men and women selected from a Brazilian population-based sample found that uncontrolled hypertensive men under drug treatment more frequently suffered chronic musculoskeletal complaints. The lack of association in women and in hypertensive men not under blood pressure drug treatment implies that hypertension is not a risk or protective factor against musculoskeletal complaints [14]. However, a long-term Finnish industrial cohort reported elevated SBP or DBP was positively associated with LBP occurrence [24]. Meanwhile, a Norwegian population-based study with prospective and cross-sectional data revealed inverse associations in both sexes between SBP or DBP and prevalence of several musculoskeletal disorders including LBP [22]. Also, Duschek et al. observed that some chronic hypotension patients reported higher pain thresholds after taking medication that elevated blood pressure [25]. On a similar note, various studies have stated that subjects consistently displayed lowered pain perception with higher blood pressure regardless of intervention type for blood pressure elevation (pharmacological means, increase in salt intake, arterial surgery) [26]. These results are not limited to musculoskeletal conditions, and while Hagen et al. reported lower occurrence of headaches in hypertension patients in a prospective study [27], 2 other studies demonstrated decreasing frequency of migraines with increasing blood pressure [28,29], and Tronvik et al. found that angina pectoris and myocardial infarction patients with hypertension presented with less pain than normotensive patients [30].

Some major strengths of our study are that our population is a large-scale, nationally representative sample, and that the health survey and examinations were conducted by trained surveyors using standardized methods. In addition, many covariates which could potentially confound the association with LBP and osteoarthritis (age, sex, household income, education, occupation, BMI, smoking status, alcohol consumption, regular moderate-intensity exercise) were considered. We included fully and selectively adjusted estimates to present our results with better reliability in sensitivity analysis, and the fact that both LBP and osteoarthritis, 2 major musculoskeletal diseases that incur considerable personal and social expenses, produced significant results is noteworthy. Investigation of lifetime LBP and osteoarthritis covering lifetime prevalence establishes the general association between hypertension and pain, and further assessment of chronic LBP and osteoarthritis-related pain over the past year confirmed that chronic pain displays similar tendencies.

The biggest limitation of our study is due to the cross-sectional design, as this method employs data collected at a specific timepoint precluding inferences of causality. Another limitation is that the information was gathered through surveys, leaving the validity and reliability of data open to question as LBP and osteoarthritis evaluation was not performed by physicians through physical assessment or diagnostic imaging but solely depended on self-report. Also, musculoskeletal pain experience was assessed with no questions pertaining to pain intensity. Investigation on antihypertensive medication was limited to regular use and use on day of survey, and failed to include medication type. There is also the possibility that false-positive patients with hypertension from physician/medical institution anxiety were included and false-negative patients with masked hypertension were excluded, as blood pressure information was gauged from on-site examination. Still, SBP and DBP was measured 3 times after a 5 minute rest, and data was compiled as the average of the 2^nd^ and 3^rd^ measurements for adjustment.

Hypalgesia related to high blood pressure was first described in animal studies manipulating blood pressure, and later in various clinical studies [13,31]. There is still considerable debate regarding physiology in the pain sensitivity-hypertension connection. hypertension may induce baroreceptor activation, resulting in decreased reaction to noxious stimuli [32]. Baroreflex arch stimulation due to increased blood pressure inhibits pain transmission at spinal and supraspinal levels, possibly through interactions with brain areas that control nociception and cardiovascular reflexes in the brainstem. Anatomically, baroreceptors are well-situated to influence neural activity in the nucleus tractus solitarius, locus coeruleus, paraventricular hypothalamus, paratrigeminal nucleus, periaqueductal grey substance and nucleus raphe magnus [26]. There is also evidence that unstimulated baroreceptive sensitivity is related to pain thresholds [33].

Endogenous opioid activity, which contributes to reduced pain sensitivity, may be involved in blood pressure-associated hypalgesia [34]. The action mechanism of endogenous opioids is related to baroreceptors [12]. It has been suggested endogenous opioids play a critical role in the interaction between resting blood pressure and pain sensitivity, and endogenous opioid dysfunction has been linked to chronic pain development [35]. However, it is unknown to what extent endogenous opioids mediate this relation as opioid blockade failed to significantly impact the blood pressure-pain sensitivity relationship [18].

Hypalgesic effects related to high blood pressure may also be explained by central nervous system dysregulation as the central nervous system modulates pain and cardiovascular function [12,36]. This view is based on the fact that the correlation between blood pressure and pain sensitivity persists even in the absence of clinical hypertension, and the effect is regarded to be ascribable to a common central mechanism involving antinociception and cardiovascular control as opposed to a specific effect pertaining to hypertension itself [22].

There is the added possibility of neurotransmitter involvement such as catecholamine [12]. Catecholamine metabolism is regulated by catechol-O-methyltransferase gene, and polymorphism of catechol-O-methyltransferase gene is surmised to modify pain response [37]. The catechol-O-methyltransferase gene may also be important in blood pressure control [38].

These results show that pain sensitivity in LBP and osteoarthritis decreases as blood pressure increases, and that the association between high blood pressure and pain sensitivity was not significant in antihypertensive drug intake. Longer duration periods of hypertension also weakened the inverse association with pain sensitivity. Our study showed a crude positive association of hypertension with LBP and osteoarthritis. When adjusted for age and sex the direction of association was changed for LBP and attenuated for osteoarthritis. Though data is not presented, separate analyses on sex and age were performed and observed for interactions, showing that age asserted the biggest influence. It can be conjectured that this is due to the fact that lifetime and chronic prevalence of musculoskeletal pain increases with age. Interactions between sex and hypertension were also tested, but tendencies differed in LBP and osteoarthritis. LBP prevalence showed tendencies toward non-association in women, and that of osteoarthritis toward non-association in men. Previous studies have also reported gender difference in associations between prevalence of hypertension and chronic disease [14], indicating a need for investigations by gender and pain complaints, and the present study would also have benefited from further stratified analyses by sex.

In addition, it is still unclear how hypertension and hypertensive medicine influence sensitivity to pain. Further prospective studies are needed to determine the exact causal relationship in pain sensitivity and hypertension to effectively prevent and treat hypertensive diseases, with additional analyses covering the underlying medical causes of hypertension (essential hypertension, secondary hypertension), quantified pain scores, and duration of pain and psychological factors which may additionally affect pain sensitivity.

## Supporting Information

S1 File Associations between blood pressure and chronic LBP and chronic osteoarthritis prevalence (DOCX)
