# Supplementary material for: Association between Hypertension and the Prevalence of Low Back Pain and Osteoarthritis in Koreans: A Cross-Sectional Study
Source: PLoS One. 2015 Sep 22;10(9):e0138790. doi: 10.1371/journal.pone.0138790 (PMC4578861; doi:10.1371/journal.pone.0138790)
Supplement: S1 File — (DOCX) [file pone.0138790.s001.docx]

Table A. Associations Between Blood Pressure Levels and Low Back Pain or Osteoarthritis in Korean Surveyees of KNHANES IV aged ≥20 years^a^

|  |  |  |  | Crude | | |  | Adjusted for age and sex | | |  | Fully adjusted^b^ | | |  | Selectively adjusted^c^ | | |
| --- | --- | --- | --- | --- | --- | --- | --- | --- | --- | --- | --- | --- | --- | --- | --- | --- | --- | --- |
|  |  | N (case) |  | OR | 95% CI | *P* |  | OR | 95% CI | *P* |  | OR | 95% CI | *P* |  | OR | 95% CI | *P* |
| Low back pain^d^ | |  |  |  |  |  |  |  |  |  |  |  |  |  |  |  |  |  |
|  | Normal | 7,804 (996) |  | 1.00 |  |  |  | 1.00 |  |  |  | 1.00 |  |  |  | 1.00 |  |  |
|  | Prehypertension | 4,165 (596) |  | 1.14 | 1.00, 1.30 | 0.0577 |  | 0.97 | 0.84, 1.12 | 0.66 |  | 0.90 | 0.77, 1.06 | 0.2057 |  | 0.92 | 0.79, 1.08 | 0.2959 |
|  | Hypertension | 5,060 (1,110) |  | 1.80 | 1.59, 2.04 | <0.0001 |  | 0.93 | 0.81, 1.07 | 0.3177 |  | 0.85 | 0.73, 0.98 | 0.0292 |  | 0.88 | 0.76, 1.02 | 0.0829 |
|  |  |  |  |  |  |  |  |  |  |  |  |  |  |  |  |  |  |  |
| Osteoarthritis^e^ | |  |  |  |  |  |  |  |  |  |  |  |  |  |  |  |  |  |
|  | Normal | 7,804 (569) |  | 1.00 |  |  |  | 1.00 |  |  |  | 1.00 |  |  |  | 1.00 |  |  |
|  | Prehypertension | 4,165 (496) |  | 1.61 | 1.38, 1.87 | <0.0001 |  | 1.16 | 0.98, 1.37 | 0.091 |  | 0.94 | 0.78, 1.13 | 0.5066 |  | 0.99 | 0.83, 1.17 | 0.8914 |
|  | Hypertension | 5,060 (1,085) |  | 3.69 | 3.24, 4.20 | <0.0001 |  | 1.29 | 1.12, 1.49 | 0.0006 |  | 0.98 | 0.83, 1.14 | 0.7569 |  | 1.00 | 0.86, 1.16 | 0.9903 |

CI, confidence interval; KNHANES, Korean National Health and Nutrition Examination Survey; OR, odds ratio.

^a^ Hypertension diagnosis was made when patients met international standards (SBP≥140 mmHg or DBP≥90 mmHg) or were already on medication.

^b^ Adjusted for age, sex, household income, education, occupation, BMI, smoking, alcohol consumption and exercise patterns.

^c^ Adjusted for age, sex, education, occupation, BMI, alcohol consumption and exercise patterns in low back pain, and age, sex, education, BMI and exercise patterns in osteoarthritis. Backward elimination method was used with *P* <0.05 regarded to be significant.

^d^ Lifetime low back pain: any previous experience of low back pain

^e^ Lifetime osteoarthritis: any previous experience of osteoarthritis

Table B. Associations Between Systolic and Diastolic Blood Pressure and Low Back Pain or Osteoarthritis in Korean Surveyees of KNHANES IV aged ≥20 years^a^

|  |  |  |  |  | Crude | | |  | Adjusted for age and sex | | |  | Fully adjusted^b^ | | |  | Selectively adjusted^c^ | | |
| --- | --- | --- | --- | --- | --- | --- | --- | --- | --- | --- | --- | --- | --- | --- | --- | --- | --- | --- | --- |
|  | Variable |  | N (case) |  | OR | 95% CI | *P* |  | OR | 95% CI | *P* |  | OR | 95% CI | *P* |  | OR | 95% CI | *P* |
| Low back pain^d^ | |  |  |  |  |  |  |  |  |  |  |  |  |  |  |  |  |  |  |
|  | SBP | <120 | 9,876 (1,260) |  | 1.00 |  |  |  | 1.00 |  |  |  | 1.00 |  |  |  | 1.00 |  |  |
|  |  | <140 | 5,118 (944) |  | 1.51 | 1.34, 1.69 | <0.0001 |  | 1.06 | 0.94, 1.21 | 0.3512 |  | 0.99 | 0.86, 1.13 | 0.8327 |  | 1.00 | 0.87, 1.15 | 0.9965 |
|  |  | ≥140 | 2,110 (505) |  | 2.06 | 1.75, 2.42 | <0.0001 |  | 0.97 | 0.81, 1.15 | 0.7195 |  | 0.86 | 0.72, 1.03 | 0.1036 |  | 0.90 | 0.76, 1.07 | 0.2418 |
|  |  |  |  |  |  |  |  |  |  |  |  |  |  |  |  |  |  |  |  |
|  | DBP | <80 | 10,536 (1,707) |  | 1.00 |  |  |  | 1.00 |  |  |  | 1.00 |  |  |  | 1.00 |  |  |
|  |  | <90 | 4,464 (716) |  | 0.95 | 0.84, 1.07 | 0.3861 |  | 0.89 | 0.78, 1.01 | 0.0628 |  | 0.86 | 0.75, 0.98 | 0.0235 |  | 0.87 | 0.76, 0.99 | 0.0378 |
|  |  | ≥90 | 2,104 (286) |  | 0.79 | 0.67, 0.93 | 0.0047 |  | 0.74 | 0.62, 0.88 | 0.0006 |  | 0.67 | 0.56, 0.80 | <0.0001 |  | 0.68 | 0.57, 0.81 | <0.0001 |
|  |  |  |  |  |  |  |  |  |  |  |  |  |  |  |  |  |  |  |  |
| Osteoarthritis ^e^ | |  |  |  |  |  |  |  |  |  |  |  |  |  |  |  |  |  |  |
|  | SBP | <120 | 9,876 (852) |  | 1.00 |  |  |  | 1.00 |  |  |  | 1.00 |  |  |  | 1.00 |  |  |
|  |  | <140 | 5,118 (844) |  | 2.10 | 1.85, 2.38 | <0.0001 |  | 1.08 | 0.94, 1.25 | 0.2618 |  | 0.87 | 0.75, 1.02 | 0.0835 |  | 0.91 | 0.79, 1.05 | 0.1815 |
|  |  | ≥140 | 2,110 (467) |  | 3.30 | 2.84, 3.82 | <0.0001 |  | 0.91 | 0.77, 1.08 | 0.2662 |  | 0.75 | 0.62, 0.90 | 0.0020 |  | 0.77 | 0.65, 0.91 | 0.0020 |
|  |  |  |  |  |  |  |  |  |  |  |  |  |  |  |  |  |  |  |  |
|  | DBP | <80 | 10,536 (1,271) |  | 1.00 |  |  |  | 1.00 |  |  |  | 1.00 |  |  |  | 1.00 |  |  |
|  |  | <90 | 4,464 (635) |  | 1.19 | 1.05, 1.34 | 0.0065 |  | 1.08 | 0.95, 1.24 | 0.2499 |  | 0.92 | 0.80, 1.07 | 0.2799 |  | 0.93 | 0.81, 1.07 | 0.3088 |
|  |  | ≥90 | 2,104 (257) |  | 1.04 | 0.88, 1.24 | 0.6564 |  | 0.99 | 0.82, 1.20 | 0.9206 |  | 0.77 | 0.63, 0.94 | 0.0103 |  | 0.81 | 0.66, 0.98 | 0.0308 |

CI, confidence interval; DBP, diastolic blood pressure; KNHANES, Korean National Health and Nutrition Examination Survey; OR, odds ratio; SBP, systolic diastolic blood pressure.

^a^ Individuals with SBP and DBP measurements

^b^ Adjusted for age, sex, household income, education, occupation, BMI, smoking, alcohol consumption and exercise patterns.

^c^ Adjusted for age, sex, education, occupation, alcohol consumption and exercise patterns in association between SBP and low back pain, and age, sex, education, occupation, BMI, alcohol consumption and exercise patterns in that between DBP and low back pain. Adjusted for age, sex, education, BMI and exercise patterns in associations between SBP and DBP with osteoarthritis. Backward elimination method was used with *P* <0.05 regarded to be significant.

^d^ Lifetime low back pain: any previous experience of low back pain

^e^ Lifetime osteoarthritis: any previous experience of osteoarthritis

Table C. Associations Between Systolic and Diastolic Blood Pressure and Low Back Pain or Osteoarthritis in Korean Surveyees of KNHANES IV with Hypertensive Drug Use aged ≥20 years^a^

|  |  |  |  |  | Crude | | |  | Adjusted for age and sex | | |  | Fully adjusted^b^ | | |  | Selectively adjusted^c^ | | |
| --- | --- | --- | --- | --- | --- | --- | --- | --- | --- | --- | --- | --- | --- | --- | --- | --- | --- | --- | --- |
|  | Variable |  | N (case) |  | OR | 95% CI | *P* |  | OR | 95% CI | *P* |  | OR | 95% CI | *P* |  | OR | 95% CI | *P* |
| Low back pain^d^ | |  |  |  |  |  |  |  |  |  |  |  |  |  |  |  |  |  |  |
|  | SBP | <120 | 673 (154) |  | 1.00 |  |  |  | 1.00 |  |  |  | 1.00 |  |  |  | 1.00 |  |  |
|  |  | <140 | 1,438 (372) |  | 1.22 | 0.93, 1.59 | 0.1505 |  | 1.18 | 0.89, 1.55 | 0.2511 |  | 1.18 | 0.88, 1.60 | 0.2772 |  | 1.17 | 0.87, 1.57 | 0.3104 |
|  |  | ≥140 | 903 (271) |  | 1.51 | 1.12, 2.03 | 0.0075 |  | 1.30 | 0.95, 1.77 | 0.1038 |  | 1.15 | 0.82, 1.61 | 0.4058 |  | 1.20 | 0.86, 1.66 | 0.2854 |
|  |  |  |  |  |  |  |  |  |  |  |  |  |  |  |  |  |  |  |  |
|  | DBP | <80 | 1,376 (410) |  | 1.00 |  |  |  | 1.00 |  |  |  | 1.00 |  |  |  | 1.00 |  |  |
|  |  | <90 | 1,075 (273) |  | 0.70 | 0.56, 0.88 | 0.0019 |  | 0.97 | 0.77, 1.24 | 0.8316 |  | 0.90 | 0.70, 1.17 | 0.4383 |  | 0.97 | 0.76, 1.25 | 0.8170 |
|  |  | ≥90 | 563 (114) |  | 0.54 | 0.41, 0.72 | <0.0001 |  | 0.91 | 0.67, 1.22 | 0.5099 |  | 0.79 | 0.57, 1.09 | 0.1428 |  | 0.84 | 0.62, 1.15 | 0.2847 |
|  |  |  |  |  |  |  |  |  |  |  |  |  |  |  |  |  |  |  |  |
| Osteoarthritis^e^ | |  |  |  |  |  |  |  |  |  |  |  |  |  |  |  |  |  |  |
|  | SBP | <120 | 673 (197) |  | 1.00 |  |  |  | 1.00 |  |  |  | 1.00 |  |  |  | 1.00 |  |  |
|  |  | <140 | 1,438 (368) |  | 0.83 | 0.66, 1.04 | 0.1132 |  | 0.76 | 0.59, 0.97 | 0.0278 |  | 0.71 | 0.54, 0.95 | 0.0192 |  | 0.71 | 0.55, 0.92 | 0.0091 |
|  |  | ≥140 | 903 (258) |  | 0.88 | 0.69, 1.14 | 0.3368 |  | 0.69 | 0.53, 0.90 | 0.0068 |  | 0.64 | 0.47, 0.86 | 0.0031 |  | 0.65 | 0.49, 0.86 | 0.0023 |
|  |  |  |  |  |  |  |  |  |  |  |  |  |  |  |  |  |  |  |  |
|  | DBP | <80 | 1,376 (424) |  | 1.00 |  |  |  | 1.00 |  |  |  | 1.00 |  |  |  | 1.00 |  |  |
|  |  | <90 | 1,075 (280) |  | 0.70 | 0.56, 0.87 | 0.0014 |  | 1.00 | 0.78, 1.28 | 0.998 |  | 0.86 | 0.66, 1.13 | 0.2785 |  | 0.91 | 0.71, 1.17 | 0.4536 |
|  |  | ≥90 | 563 (119) |  | 0.52 | 0.40, 0.68 | <0.0001 |  | 0.91 | 0.68, 1.21 | 0.5155 |  | 0.74 | 0.54, 1.01 | 0.0609 |  | 0.82 | 0.61, 1.10 | 0.1807 |

CI, confidence interval; DBP, diastolic blood pressure; KNHANES, Korean National Health and Nutrition Examination Survey; OR, odds ratio; SBP, systolic diastolic blood pressure.

^a^ Individuals with hypertensive medicine use of ≥15 doses per month or hypertensive medicine use on day of survey (N=3014)

^b^ Adjusted for age, sex, household income, education, occupation, BMI, smoking, alcohol consumption and exercise patterns.

^c^ Adjusted for age, sex, education, occupation and BMI in low back pain, and age, sex, education and BMI in osteoarthritis. Backward elimination method was used with *P* <0.05 regarded to be significant.

^d^ Lifetime low back pain: any previous experience of low back pain

^e^ Lifetime osteoarthritis: any previous experience of osteoarthritis

Table D. Associations Between Hypertension Duration Period and Low Back Pain or Osteoarthritis in Korean Surveyees of KNHANES IV aged ≥20 years^a^

|  |  |  |  |  | Crude | | |  | Adjusted for age and sex | | |  | Fully adjusted^b^ | | |  | Selectively adjusted^c^ | | |
| --- | --- | --- | --- | --- | --- | --- | --- | --- | --- | --- | --- | --- | --- | --- | --- | --- | --- | --- | --- |
|  | Variable |  | N (case) |  | OR | 95% CI | *P* |  | OR | 95% CI | *P* |  | OR | 95% CI | *P* |  | OR | 95% CI | *P* |
| Low back pain^d^ | |  |  |  |  |  |  |  |  |  |  |  |  |  |  |  |  |  |  |
|  | HTN duration period^e^ | | |  |  |  |  |  |  |  |  |  |  |  |  |  |  |  |  |
|  |  | ≤3 | 1,156 (265) |  | 1.00 |  |  |  | 1.00 |  |  |  | 1.00 |  |  |  | 1.00 |  |  |
|  |  | ≤5 | 547 (135) |  | 1.12 | 0.84, 1.47 | 0.4442 |  | 0.99 | 0.73, 1.33 | 0.9191 |  | 1.00 | 0.72, 1.40 | 0.9872 |  | 1.01 | 0.73, 1.39 | 0.9577 |
|  |  | ≤10 | 765 (199) |  | 1.24 | 0.97, 1.59 | 0.0939 |  | 1.08 | 0.82, 1.42 | 0.5688 |  | 1.08 | 0.81, 1.44 | 0.5868 |  | 1.08 | 0.82, 1.44 | 0.5785 |
|  |  | >10 | 699 (214) |  | 1.73 | 1.32, 2.27 | <0.0001 |  | 1.15 | 0.85, 1.55 | 0.3576 |  | 1.27 | 0.92, 1.77 | 0.148 |  | 1.23 | 0.90, 1.70 | 0.1975 |
|  |  | *P* for trend |  |  |  |  | <0.0001 |  |  |  | 0.3126 |  |  |  | 0.1463 |  |  |  | 0.189 |
|  |  |  |  |  |  |  |  |  |  |  |  |  |  |  |  |  |  |  |  |
| Osteoarthritis^f^ | |  |  |  |  |  |  |  |  |  |  |  |  |  |  |  |  |  |  |
|  | HTN duration period^e^ | |  |  |  |  |  |  |  |  |  |  |  |  |  |  |  |  |  |
|  |  | ≤3 | 1,156 (266) |  | 1.00 |  |  |  | 1.00 |  |  |  | 1.00 |  |  |  | 1.00 |  |  |
|  |  | ≤5 | 547 (128) |  | 1.01 | 0.75, 1.35 | 0.9632 |  | 0.86 | 0.62, 1.19 | 0.3693 |  | 0.85 | 0.60, 1.21 | 0.3729 |  | 0.86 | 0.61, 1.20 | 0.3605 |
|  |  | ≤10 | 765 (186) |  | 1.19 | 0.91, 1.57 | 0.2076 |  | 1.03 | 0.76, 1.38 | 0.8532 |  | 0.87 | 0.63, 1.21 | 0.4152 |  | 0.98 | 0.73, 1.34 | 0.9193 |
|  |  | >10 | 699 (251) |  | 2.17 | 1.70, 2.77 | <0.0001 |  | 1.45 | 1.11, 1.89 | 0.0071 |  | 1.37 | 1.02, 1.84 | 0.0346 |  | 1.37 | 1.03, 1.82 | 0.0313 |
|  |  | *P* for trend |  |  |  |  | <0.0001 |  |  |  | 0.0139 |  |  |  | 0.1223 |  |  |  | 0.0544 |

CI, confidence interval; KNHANES, Korean National Health and Nutrition Examination Survey; OR, odds ratio.

^a^ Individuals with data for time of HTN diagnosis (N=3167)

^b^ Adjusted for age, sex, household income, education, occupation, BMI, smoking, alcohol consumption and exercise patterns.

^c^ Adjusted for age, sex, education, occupation and BMI in low back pain, and age, sex, education and BMI in osteoarthritis. Backward elimination method was used with *P* <0.05 regarded to be significant.

^d^ Lifetime low back pain: any previous experience of low back pain

^e^ Duration period was classified as quartiles.

^f^ Lifetime osteoarthritis: any previous experience of osteoarthritis
